# Potential Impacts of Different Occupational Outdoor Heat Exposure Thresholds among Washington State Crop and Construction Workers and Implications for Other Jurisdictions

**DOI:** 10.3390/ijerph191811583

**Published:** 2022-09-14

**Authors:** John C. Flunker, Christopher Zuidema, Jihoon Jung, Edward Kasner, Martin Cohen, Edmund Seto, Elena Austin, June T. Spector

**Affiliations:** 1 Department of Environmental and Occupational Health Sciences, Hans Rosling Center for Population Health, University of Washington, Seattle, WA 98195, USA; 2 Safety & Health Assessment and Research for Prevention (SHARP) Program, Washington State Department of Labor and Industries, Olympia, WA 98504, USA

**Keywords:** occupational heat exposure, heat rule, policy, outdoor workers, crop and construction employment, maximum temperature exceedances, Washington State

## Abstract

Occupational heat exposure is associated with substantial morbidity and mortality among outdoor workers. We sought to descriptively evaluate spatiotemporal variability in heat threshold exceedances and describe potential impacts of these exposures for crop and construction workers. We also present general considerations for approaching heat policy-relevant analyses. We analyzed county-level 2011–2020 monthly employment (Bureau of Labor Statistics Quarterly Census of Employment and Wages) and environmental exposure (Parameter-elevation Relationships on Independent Slopes Model (PRISM)) data for Washington State (WA), USA, crop (North American Industry Classification System (NAICS) 111 and 1151) and construction (NAICS 23) sectors. Days exceeding maximum daily temperature thresholds, averaged per county, were linked with employment estimates to generate employment days of exceedances. We found spatiotemporal variability in WA temperature threshold exceedances and crop and construction employment. Maximum temperature exceedances peaked in July and August and were most numerous in Central WA counties. Counties with high employment and/or high numbers of threshold exceedance days, led by Yakima and King Counties, experienced the greatest total employment days of exceedances. Crop employment contributed to the largest proportion of total state-wide employment days of exceedances with Central WA counties experiencing the greatest potential workforce burden of exposure. Considerations from this analysis can help inform decision-making regarding thresholds, timing of provisions for heat rules, and tailoring of best practices in different industries and areas.

## 1. Introduction

Occupational heat exposure is associated with substantial morbidity and mortality, including among agricultural and construction workers [1,2,3]. Heat exposure can cause heat-related illnesses (HRIs), including heat stroke, which can be fatal, and heat exhaustion, heat syncope (fainting), rhabdomyolysis, and heat cramps. Data from the United States (US) Bureau of Labor Statistics (BLS) Census of Fatal Occupational Injuries (CFOI) from 2000 to 2010 indicate that agricultural workers had a yearly average heat-related fatality rate of 3.1 per 1,000,000 workers and the highest risk of heat-related deaths compared to all other industries (rate ratio 35.2 (95% confidence interval 26.3–47.0)) [1]. Construction workers had the second highest risk of heat-related death (rate ratio 13.0 (95% confidence interval 10.1–16.7)), with a yearly average fatality rate of 1.1 per 1,000,000 workers [1]. In an analysis of accepted Washington (WA) State Fund HRI workers’ compensation claims, public administration, agriculture, forestry, and fishing (AFF) and construction sectors had the highest third-quarter (July–September) rates of HRI (131.3, 102.6, and 70 HRI claims per 100,000 full-time employees (FTE), respectively) [2]. HRIs are likely underestimated, as less severe work-related HRIs may not be identified or reported [2]. Occupational heat exposure is also associated with acute kidney injury, including among agricultural workers [4,5], and traumatic injuries, including among agricultural workers and outdoor construction workers [6,7,8,9].

Occupational health and safety rules intended to protect outdoor workers from heat exposure have been developed in several states in the US. California (CA) and WA have implemented outdoor heat exposure rules in 2005 and 2008, respectively. As of 2022, WA is in the process of updating its permanent outdoor heat rule [10], and Oregon (OR) has recently adopted an occupational heat rule that applies in indoor and outdoor settings [11]. Maryland [12] and Nevada [13] are also pursuing heat rules that include provisions for outdoor workers. The US Occupational Safety & Health Administration (OSHA) has published an Advanced Notice of Proposed Rulemaking for heat [14]. Occupational health and safety rulemaking considers feasibility in addition to science. For example, to adopt permanent rules in WA, Administrative Procedure Act procedures must be followed, which include a cost–benefit analysis to determine whether the probable economic benefits of the rule are greater than its probable costs [15]. Information about worker exposure and health impacts is helpful in characterizing the potential costs and benefits associated with policies.

In contrast to rules, non-enforceable occupational health and safety guidelines provide science-based heat-stress risk assessment and management recommendations. As defined by American Conference of Governmental Industrial Hygienists (ACGIH), heat stress is the total heat load to which a worker may be exposed from the combined contributions of metabolic heat from physical work, environmental factors, and clothing requirements [16]. To define occupational exposure limits, the ACGIH and National Institute for Occupational Safety and Health (NIOSH) heat stress guidelines use the Wet Bulb Globe Temperature (WBGT), which takes into account dry air temperature, humidity, wind, and solar radiation. WBGT requires special measurement instrumentation or can be estimated from certain weather parameters [17], though these input parameters may not be readily available from all data sources. Forecasted WBGT is usually not accessible for work planning. The Heat Index is a “real feel” metric that is a function of temperature and relative humidity [18] and has been used when WBGT is not practical or available [19,20,21]. However, the heat index does not account for the effects of wind or solar radiation. Temperature and heat index are more straight-forward metrics for the general public, employers, and workers and can be forecasted for work planning. No existing US State occupational health & safety rules define enforceable heat thresholds using WBGT.

ACGIH enumerates recommendations for general controls at an Action Limit, intended for workers who are not acclimatized to the heat [16]. General controls include training, hydration, and other elements. More intensive job-specific administrative and engineering controls are recommended at higher threshold limit values, intended for workers acclimatized to heat. Threshold limit values are approximately 2.5–3.0 °C higher than action limits, depending on work cycles and work intensity [16]. Acclimatization consists of gradual physiological adaptations that occur with exposure to work in the heat [22,23]. These adaptations include increased sweating efficiency and blood flow to the skin, which supports evaporative cooling, and the ability to perform work at lower core body temperatures and heart rates. Acclimatization can take up to two weeks to develop, with a substantial amount of adaptation occurring within the first four to five days. NIOSH recommends four-to-five-day acclimatization schedules that consist of gradually increasing exposure time in hot environments by about 20% on each successive day, depending on level of experience [22]. However, acclimatization can be lost after about a week away from working in the heat. Acclimatization is therefore particularly important for new workers, workers returning from a prolonged absence, and workers exposed to sudden increases in heat.

Most existing heat rules stipulate the level of heat exposure at or above which rule requirements apply (e.g., “trigger”) and the level of heat exposure at which additional protections are required (e.g., “high heat threshold”). The trigger in the current WA permanent heat rule is an air temperature of 89 °F (31.7 °C) for workers wearing regular work clothes [24,25] and an air temperature of 80 °F (26.7 °C) in the CA heat rule [26] and 80 °F (26.7 °C) heat index (HI) in the OR heat rule [11]. Notably, a HI of 80 °F (26.7 °C) is approximately equal to a dry air temperature of 80 °F (26.7 °C) at a dew point of 50 °F (10.0 °C). For WA’s current permanent outdoor heat rule, humidity considerations were integrated into the development of the temperature triggers. The dew points of four WA cities (Vancouver, Seattle, Yakima, and Spokane) in the summer of 2007 were considered, and it was determined that a “dew point of 50 °F (10.0 °C) was the average within two standard deviations for [WA]…” [27]. The dew point is an appropriate metric for assessing humidity, as it is the temperature to which air must be cooled to become saturated with water vapor, assuming constant air pressure, and is therefore a more absolute measure than relative humidity, which depends on the dew point and temperature. A dew point of 50 °F (10.0 °C) and assumptions of moderate metabolic rate work, unacclimatized workers, and work in the sun with different clothing ensembles were used to determine the corresponding dry air temperatures for the WBGT-based ACGIH heat-stress action limits, and these became the WA heat rule trigger temperatures [27]. In the CA heat rule, the high heat threshold is an air temperature of 95 °F (35.0 °C) [26], and in the OR heat rule, the high heat threshold is a HI of 90 °F (32.2 °C) [11]. 

The CA heat rule has an additional exposure designation of “heat wave,” defined as any day in which the predicted high temperature for the day will be at least 80 °F (26.7 °C) and at least 10 °F higher than the average high daily temperature in the preceding five days [26]. During heat wave days, additional acclimatization provisions are required. The distinction between a “heat wave” day and exceedances of thresholds is that a “heat wave,” as defined by the CA heat rule [26], captures changes in temperature, relative to previous days, to which workers may not have had sufficient time to become acclimatized.

The intensity and duration of heat events is projected to increase in the future [28]. In the summer of 2021, the Pacific Northwest experienced a “heat dome” event, in which high pressure circulation in the atmosphere trapped heat, resulting in an unprecedented heat wave and an increase in WA HRI workers’ compensation claims [29]. An attribution analysis indicated that this event would have been virtually impossible without climate change and is estimated to occur roughly every 5 to 10 years with 2 °C of global warming [30]. Protections and adaptations of existing heat rules are needed for workers, including agricultural and construction workers, who perform physical labor in the sun (e.g., crop workers, roofing workers), perform time-sensitive tasks (e.g., harvesting, pouring concrete), and are new or exposed to heat waves to which they may not be acclimatized [2]. The aims of this study were to spatiotemporally describe and compare: (1) exceedances of different existing heat rule thresholds that trigger workplace heat controls; and (2) crop and construction populations at risk of exposure to hot working conditions that are at or in excess of temperature thresholds (employment days), using historical weather and employment data in WA. Our primary analysis focused on air temperature thresholds, and our secondary analyses focused on heat waves. The ultimate goal of our analyses is to inform decisions about heat rulemaking and rule implementation and to provide examples of methods that could be used in other states and jurisdictions to support heat rule decision-making. 

## 2. Materials and Methods

### 2.1. Study Area, Populations, and Setting

We analyzed 2011–2020 Washington State, US, agriculture and construction employment data and environmental data at the county level for all WA counties. This time period did not include the 2021 Pacific Northwest “heat dome” event. We focused on construction and crop sectors, as these groups have high HRI rates [2], large proportions of outdoor workers, and different geographical distributions, and thus, different potential exposures to environmental heat across WA. In WA, particularly in rural Central WA, agriculture is a major component of the economy [31]. Top crops include apples, grapes, hops, and cherries [32], which are prepared and harvested from approximately May to November [33]. WA agricultural workers include seasonal workers and US H-2A guest workers from other countries, who are hired on temporary work permits by agricultural employers [34]. Commercial and residential construction employment has been generally increasing in WA, with seasonal trends peaking in the summer months in urban areas of Eastern and Western WA [35]. Public administration also has high HRI worker compensation claim rates in WA, with the highest third-quarter public administration HRI rates among fire protection [2]. Fire protection involves different considerations for heat exposure, including point sources of heat and specialized personal protective equipment, and is addressed outside of WA general industry and agriculture heat rules. We therefore did not include public administration in this study. 

The weather in WA is variable [36]. Western WA is relatively mild, with summer maximum temperatures infrequently rising above 79 °F (26.1 °C) and frequent clouds in the winter, spring, and fall. The annual rainfall in the greater Seattle, WA, area is approximately 37 inches (94 cm), and July and August are the driest Western WA months. The greater Seattle area is surrounded by the Olympic mountains to the West, the Cascade mountains to the East, Mount Baker to the North, and Mount Rainier to the South. East of the Cascades, in Central WA, conditions are drier, with seven to nine inches (18 to 23 cm) of rain annually. Average summer high temperatures in Central WA are typically in the upper 80 to mid-90 °F (27–34 °C). We expect the contribution to HRI risk from outdoor environmental exposure to be higher in hotter areas of Central/Eastern WA and during periods of sudden change in temperature across the state, including in Western WA, when workers may not be acclimatized to heat.

### 2.2. Data Sources

Heat exposure and employment data sources are described in detail in the subsections below. In general, different data sources were selected to best address corresponding key policy-relevant questions and for primary versus secondary analyses.

### 2.3. Approach to Selecting Exposure Data Sources

First, to compare times of year and temperature thresholds across WA, we used gridded, modeled environmental data (Parameter-elevation Relationships on Independent Slopes Model (PRISM) data [37]). PRISM data are an accurate, downloadable source of gridded weather data [38]. Unlike weather station data, PRISM data have even spatial coverage but are not available in real time so are most appropriate for questions that can be addressed with historical data such as the time of year, metrics, and thresholds for heat. Once decisions about these questions are integrated into policies, employers and workers can, in general, use real-time and forecasted temperature data such as data from National Weather Service weather stations to guide best practices and compliance with rules. The HI from nearest available weather stations and forecasts for the week are also available using the NIOSH/OSHA mobile heat application; this tool does not provide information in the form of dry air temperature or WBGT [39].

Second, for certain sectors in rural areas such as agriculture, National Weather Service weather stations may not have adequate spatial coverage in the areas where workers of interest are located, may not reflect local field conditions, and may not be a trusted source of information. Our secondary heat wave analysis therefore uses an example of a weather station network, AgWeatherNet (AWN) [40], with enhanced coverage in agriculturally intensive areas of WA, relevant to crop employment. AWN is an application of sensor networks that provides real-time, local information intended for crop decision support. AWN is a trusted source of weather data and consists of a network of over 200 professional weather stations located primarily in agriculturally productive regions of Central/Eastern WA [40]. We chose AWN as an example for heat wave analyses relevant to agriculture because effective identification of heat wave days in real time by agricultural employers and workers requires local, available, and trusted sources of temperature data. 

### 2.4. Exposure Data Sources

WA county-level exposure data were derived from PRISM interpolated data [37]. PRISM was developed by the PRISM Climate Group at Oregon State University by incorporating climate observations from monitoring networks, applying quality-control measures, and developing spatial datasets at a grid resolution of 800 m, which have been filtered to 4 km pixel resolution for free downloading [41]. PRISM models consider the location, elevation, coastal proximity, topographic facet orientation, vertical atmospheric layer, topographic position, and orographic effectiveness of the terrain. We extracted the PRISM daily maximum temperature (dT_max_) in °F. Next, we summarized values across each county by computing the mean dT_max_ across all 4 × 4 km pixels within each county (polygon), which we refer to as the T_max_.

For secondary analyses of heat threshold exceedances and days meeting the CA heat wave definition, we used data from Washington State University’s AWN Program [40]. We obtained hourly temperature data in °F from AWN stations, which log data in 15 min intervals. We identified the daily maximum temperature (dT_max_) for each weather station within a county each day and averaged these values together to obtain the mean dT_max_ value for each county on each day, which we refer to as the T_max_. Using T_max_ for each day, we determined the number of heat wave days per county during the study period using the CA heat rule definition (any day in which the predicted high temperature for the day will be at least 80 °F (26.7 °C) and at least 10 °F higher than the average high daily temperature in the preceding five days) [26].

### 2.5. Employment Data Source

We obtained WA state employment data from the US Bureau of Labor Statistics (BLS) Quarterly Census of Employment and Wages (QCEW) [42] by month, county, and 6-digit North American Industry Classification System (NAICS) codes for NAICS 236, 237, and 238 (construction) and 111 (crop production) and 1151 (Support Activities for Crop Production). Most construction workers were considered to have a high or medium probability of outdoor work [35], while crop production and support activities for crop production were assumed to have a high potential for outdoor work. QCEW includes data from employers who report their employment and wages to pay unemployment insurance taxes. QCEW monthly employment data estimate industry-level employment for all full-time, part-time, or piece-rate workers, who worked during or received pay (e.g., paid sick leave, vacation, holiday) for the pay period that included the 12th day of the month. QCEW estimates jobs/employment, and since this includes full- and part-time employment, employment counts may not be the same as the number of workers. QCEW data do not include all employment. For example, unpaid family members, self-employed workers, and some farm/domestic workers are not included. BLS estimates that the QCEW covers 80 percent of farmworker employment. In WA in 2016, approximately 7% of construction workers were independent contractors [43]. In WA, NAICS 115115 (farm labor contractors) includes workers participating in the H-2A visa program [2]. Because the same employment may continue from month to month, county-level monthly average employment was calculated among the months of interest (i.e., May–September or October–April) for the 10-year study duration. 

### 2.6. Data Analysis

Using PRISM data, we developed maps of the annual average number of days per county during the study period (2011–2020) at or exceeding T_max_ of 80, 85, 89, 90, and 95 °F (26.7, 29.4, 31.7, 32.2, 35.0 °C, respectively) from May to September and October to April. These thresholds were chosen to mirror current WA, OR, and CA triggers and high heat thresholds [11,24,25,26]. May–September is the current timeframe that the WA heat rule is in effect [24,25], while OR and CA heat rules apply all year [11,26]. We also developed monthly time series plots by county of exceedance thresholds. For secondary analyses, to further examine the heat exposure and potential HRI exposure burden among crop workers in Central Washington, we developed maps of both annual average temperature exposure exceedances and the annual average number of heat wave days per county during the study period based on AWN data. 

We developed maps of the annual average total crop production and crop production support, construction, and total employment by county over the study period. We also developed monthly time-series plots by county, overlaying the number of days at or above thresholds with the percent change in employment. The percent change in employment per month was calculated by dividing each month’s employment total (numerator) by the month with the lowest employment total per county (denominator) multiplied by 100. We computed employment days of exposure by multiplying employment per NAICS industry code by days at or above thresholds for each month in a year and adding those products together to yield the total number of employment days at or above each threshold in a year. We developed maps of the annual average employment days per county during the study period (2011–2020) at or exceeding thresholds and, for secondary analyses, during heat wave days, from May to September and from October to April. 

All analyses were performed using R [44], version 4.2.0 (Vienna, Austria).

## 3. Results

### 3.1. Temperature Threshold Exceedances (PRISM)

**Figure 1:**
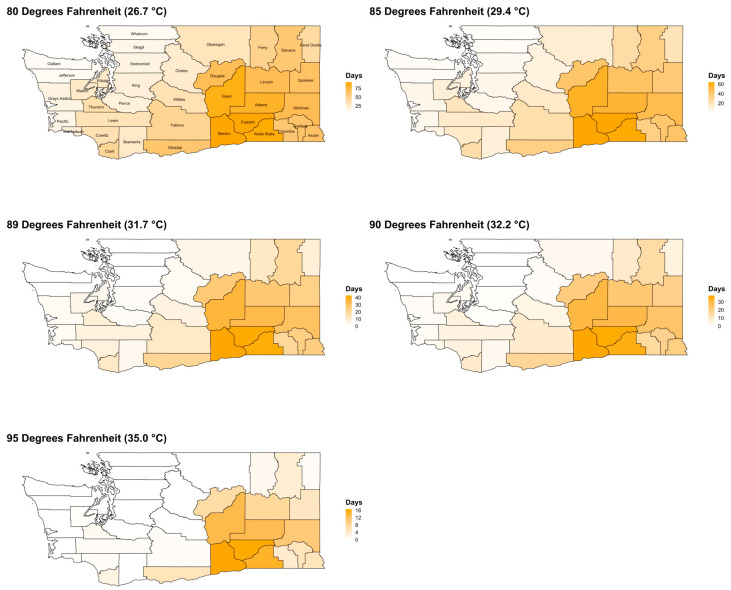
Maps of the annual average number of days from May to September at or above PRISM-derived temperature thresholds by WA county, 2011–2020. Note: Color gradient scale differs per temperature threshold map. San Juan and Island counties are not labeled.

**Figure A1:**
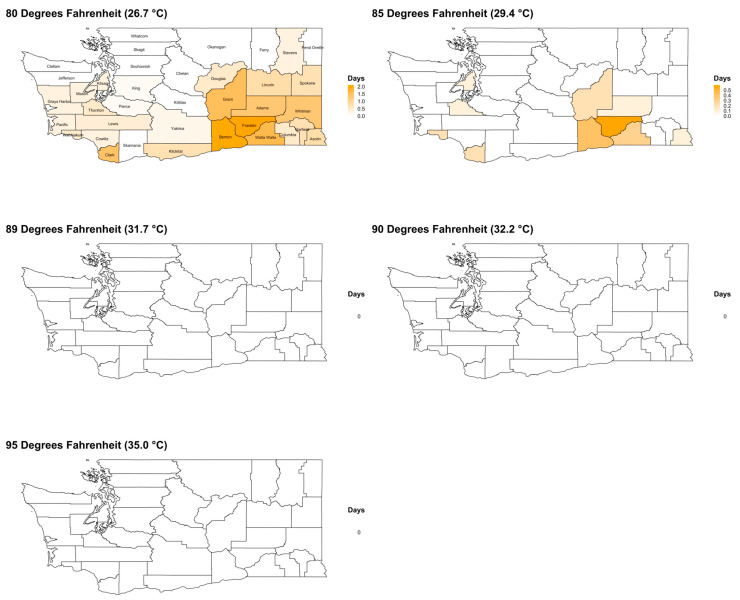
Maps of the annual average number of days from April to October at or above PRISM-derived temperature thresholds by WA county, 2011–2020. Note: Color gradient scale differs per temperature threshold map. Note: San Juan County and Island County are not labeled.

Annual average exceedances of temperature thresholds over the entire study period (2011–2020) from May to September are shown in Figure 1 and Table S1, and from October to April in Figure A1 and Table S2. The number of exceedances was generally highest in Central WA counties and lowest in coastal areas of Western WA, and the number of exceedances decreased as threshold temperatures increased. Among all WA counties from 2011 to 2020, exceedances were more likely to occur in May–September than October–April. The annual average total number of exceedance days ≥80 °F (26.7 °C) per county summed over May–September was 41.7 (SD = 26.4) while the annual average per month was 8.3 days (SD = 5.3). For the period of October–April, the annual average total number of exceedances ≥80 °F (26.7 °C) was 5.0 days (SD = 5.9) with a monthly annual average of 0.7 days (SD = 0.8). Exceedances at thresholds above 80 °F (26.7 °C) followed similar spatial patterns for both the May to September and October to April time periods, with the majority of exceedances localized to Central WA. 

**Figure 2:**
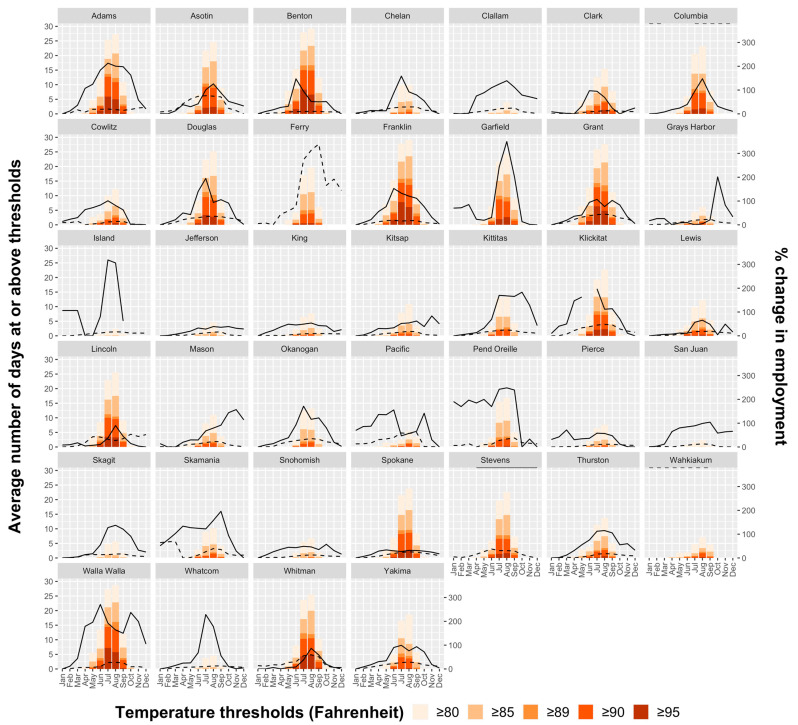
Average number of days at or above PRISM-derived temperature thresholds and employment by county and month for crop, crop support, and construction employment, 2011–2020. Dashed line: NAICS 23 (Construction); solid line: NAICS 111 (Crop production) and NAICS 1151 (Support Activities for Crop Production). Note: Month is abbreviated as the first three letters of each month.

Exceedances of temperature thresholds by month and county are shown in Figure 2. The largest annual average number of exceedance days from May to September was noted in Central WA, including Benton, Franklin, Walla Walla, Grant, Adams, and Whitman. The two counties with the maximum observed annual average of temperature threshold exceedances from May to September, Benton and Franklin, also demonstrate notable exceedances of higher thresholds. Both counties experience an annual average of approximately 90 exceedance days (18 days per month) at or above 80 °F (26.7 °C) from May to September, with the majority of these ≥85 °F (29.4 °C). Notably, almost half of the monthly average total exceedance days were at or above 90 °F (32.2 °C) in July and August for Benton and Franklin Counties. In contrast, Western WA counties such as King County demonstrated fewer exceedances at higher thresholds, with exceedances at lower thresholds primarily occurring in the May–September period in the months of July and August. Among all counties, temperature exceedances tended to be most frequent in July and August, yet temperature exceedances spanned a broader range of months in the Central WA Counties than in Western WA Counties. Exceedances between October and April occurred most frequently in the Central WA counties, all of which occurred in October and April. There were no exceedances of temperature thresholds at or above 89 °F (29.4 °C) in any county from October to April. The total number of exceedances per year among all counties from 2011 to 2020 showed no distinguishable trends over time.

### 3.2. Employment

**Figure 3:**
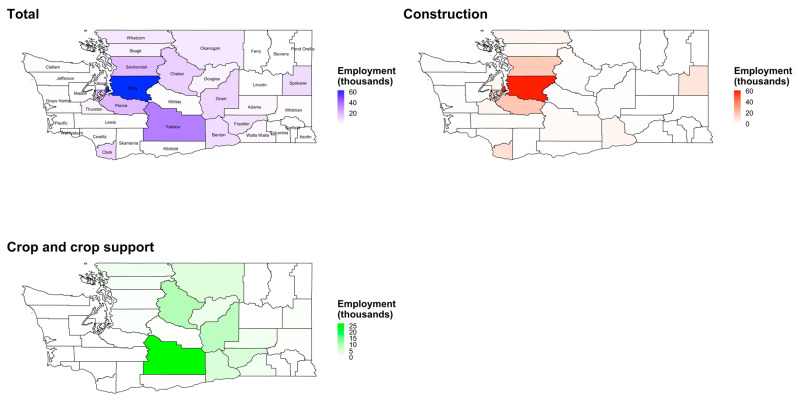
Maps of the annual average employment (thousands) for total employment, construction, and crop/crop support by county, averaged over the months of May–September, 2011–2020.

**Figure A2:**
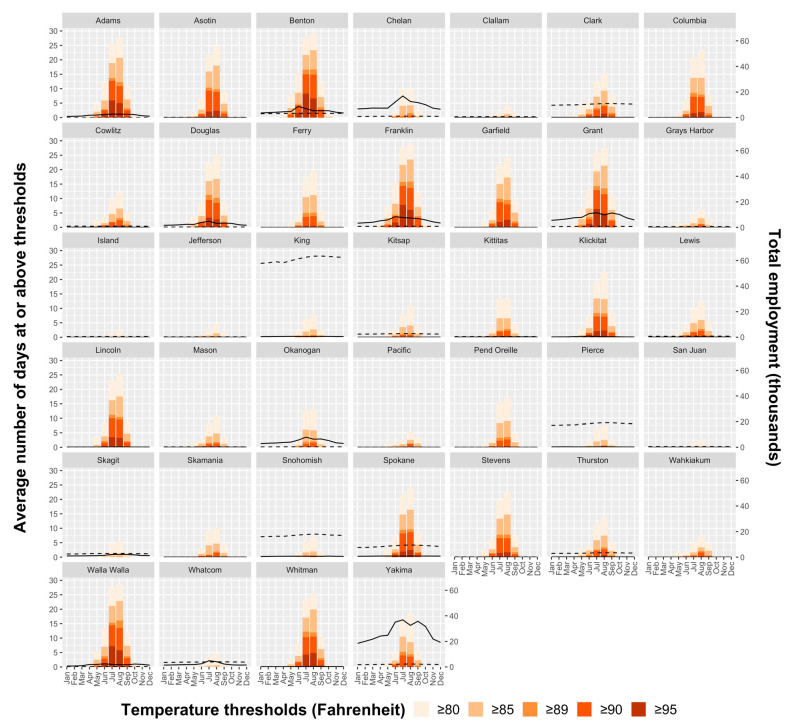
Average number of days at or above PRISM-derived temperature thresholds and employment (in thousands) by county and month for crop/crop support, and construction employment, 2011–2020. Dashed line: NAICS 23 (Construction); solid line: NAICS 111 (Crop production) and NAICS 1151 (Support Activities for Crop Production). Note: Month is abbreviated as the first three letters of each month.

Average annual total employment in thousands, averaged over the months of May to September, is shown in Figure 3 and Table S3, with maps for the entire study population and separately for construction and crop support by county, from 2011 to 2020. Overall, King County had the highest average annual monthly employment (62,800) followed by Yakima County (35,228). There was higher construction employment in urban areas of Eastern and Western WA and higher crop employment in Central WA counties. King County had the highest construction employment (62,147 average monthly employment), while Yakima County had the highest crop and crop support employment (33,012 average monthly employment). Figure 2 shows seasonal variation in employment by county and month, in terms of percent change in average monthly employment per county, with larger peaks in employment in the summer months among crop employment (e.g., in the Central WA county of Yakima) than construction employment. These peaks are also visualized in plots of employment expressed as absolute rather than percentage employment (Figure A2). 

### 3.3. Relationship between Temperature Threshold Exceedances and Crop and Construction Employment

**Figure 4:**
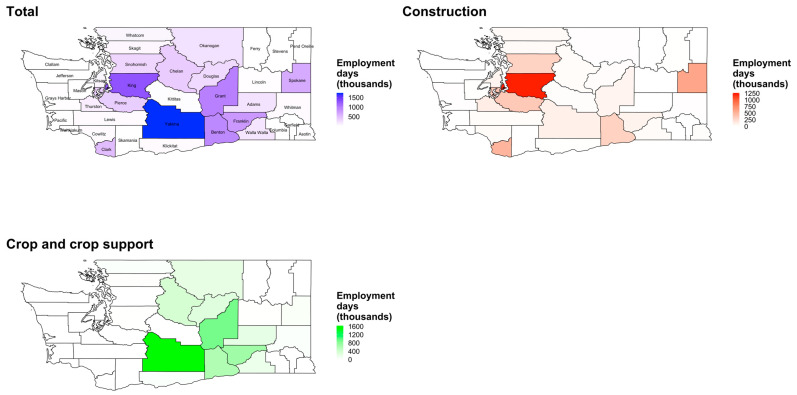
The annual average employment days (thousands) from May to September at or above PRISM-derived temperature threshold of 80 degrees Fahrenheit (26.7 °C) by county and for crop and construction employment, 2011–2020. Note: San Juan County and Island County are not labeled.

As shown in Figure 2 and Figure A2, certain counties exhibit lower numbers of threshold exceedances but high employment (e.g., King County, for construction), and certain counties experience peaks in employment with high employment percentage (relative to the lowest month of employment per county) overlapping with peaks in threshold exceedances (e.g., Yakima County, for crop and crop support). As such, these counties exhibit high employment days at or above thresholds. Figure 4 and Tables S4–S6 show the total employment days at or above a PRISM-derived temperature threshold of 80 °F (26.7 °C) by county and for crop and construction employment from May to September. We found a state-wide total yearly average of 9.46 million employment days at or above the PRISM-derived temperature threshold of 80 °F from May to September, with 5.32 million from crop and crop support employment and 4.14 million from construction employment. Counties with highest construction employment days at or above temperature thresholds include King, Spokane, Clark, Snohomish, and Pierce Counties, and counties with the highest crop employment days include Yakima, Chelan, Grant, Benton, and Franklin Counties. Though employment among all workers is highest in King County, threshold exceedances are substantial in Yakima County, and Yakima County has the highest yearly average total employment days between May and September (1.74 million days) among all workers. King county closely followed Yakima in yearly average total employment days between May and September (1.28 million days) among all workers.

**Figure A3:**
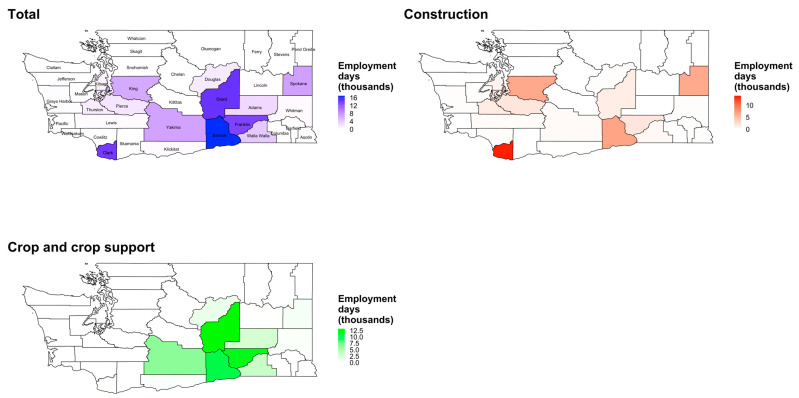
The annual average employment days (thousands) per year from October to April at or above PRISM-derived temperature threshold of 80 degrees Fahrenheit by county and for crop and construction employment, 2011–2020. Note: San Juan County and Island County are not labeled.

Figure A3 shows annual average employment days at or above a PRISM-derived temperature threshold of 80 °F (26.7 °C) by county and for crop and construction employment from October to April. Though there are not a substantial number of days exceeding 80 °F (26.7 °C) outside of May to September (Figure A1), employment continues outside May to September (Figure 2). For the October to April period, construction employment days are highest in Clark, Benton, King, and Spokane Counties, and crop employment days are highest in Grant, Franklin, Benton, and Yakima Counties (Figure A3). Among all workers, the yearly average number of employment days from October to April was greatest in Benton County (17,000 employment days). Although lower in number than the period of May to September, all employment days at risk between October and April occurred in the months of April and October. Among all counties from 2011 to 2020, crop employment contributed to 57% (5.32 million days) of the total state-wide annual average employment days (9.46 million days) at or above the PRISM-derived temperature threshold of 80 °F (26.7 °C).

### 3.4. Secondary Analyses 

#### 3.4.1. AgWeatherNet (AWN)

##### AWN Temperature Exposures

**Figure A4:**
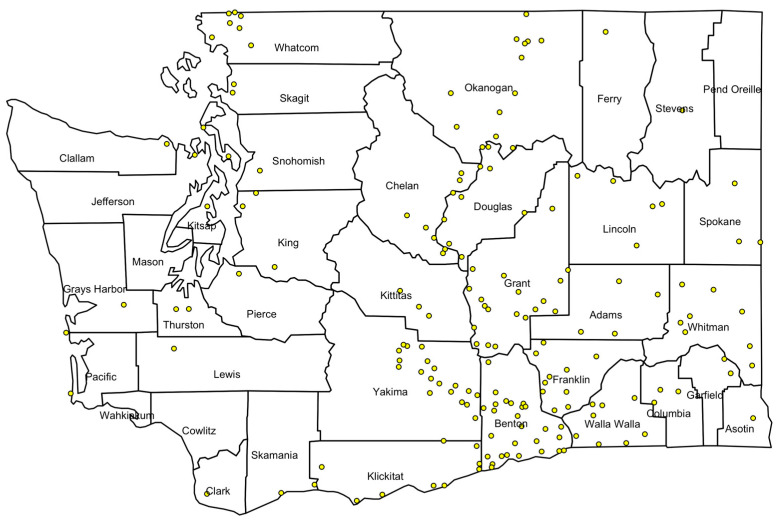
Map of WA AgWeatherNet (AWN) station locations. Yellow outlined dots represent AWN station locations. Note: San Juan County and Island County are not labeled.

**Figure A5:**
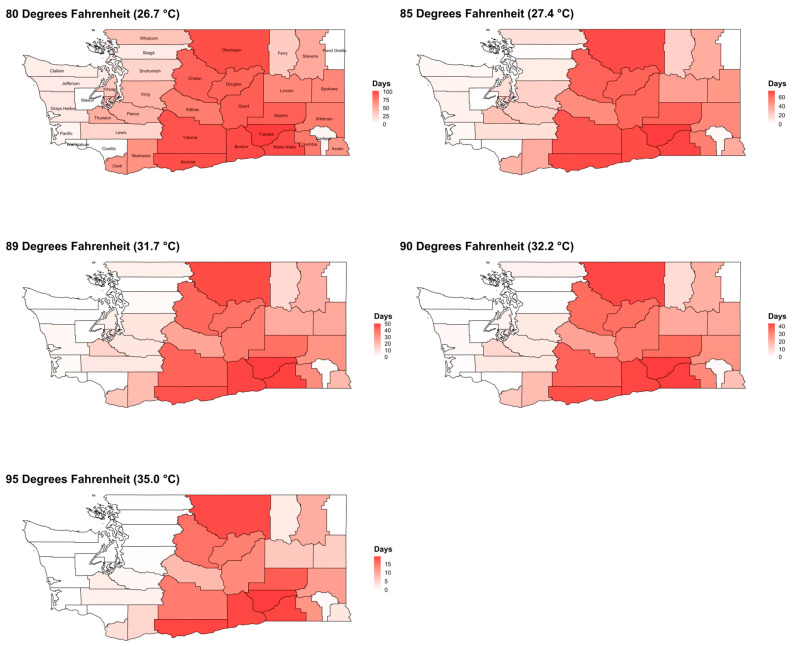
Maps of the annual average number of days from May to September at or above AWN-derived temperature thresholds by WA county, 2011–2020. Note: Color scale differs per temperature threshold. San Juan County and Island County are not labeled.

**Figure A6:**
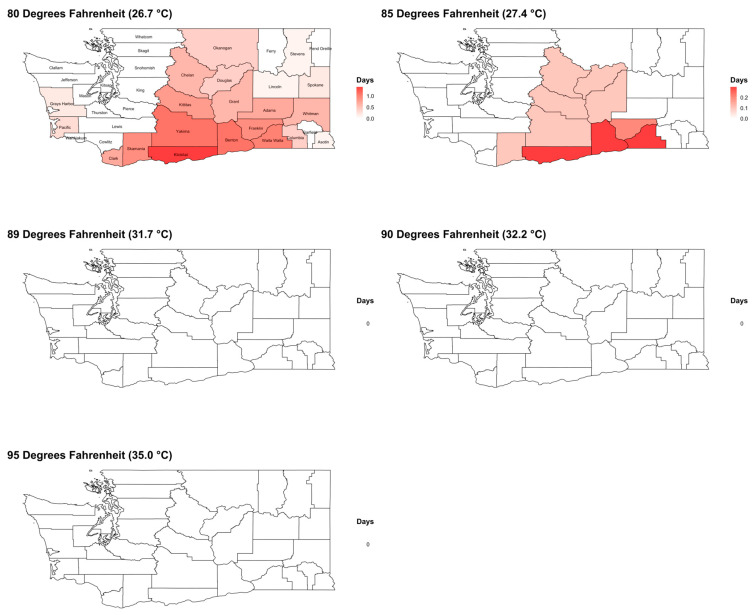
Maps of the annual average number of days from October–April at or above AWN-derived temperature thresholds by WA county, 2011–2020. Note: Color scale differs per temperature threshold. San Juan County and Island County are not labeled.

AWN stations tend to be positioned in close proximity to agriculturally active areas within the Central WA counties and exist in all but five WA counties state-wide (San Juan, Mason, Wahkiakum, Cowlitz, and Pend Oreile) (Figure A4). Spatial patterns of AWN-derived annual average heat threshold exceedances are shown in Figure A5 and Figure A6 and Tables S7 and S8, demonstrating a similar spatial and temporal pattern to that of PRISM-derived threshold exceedances. Yearly AWN-derived temperature threshold exceedances for the months of May–September were highest in Central WA counties, led by Franklin, Benton, and Walla Walla counties. The majority of exceedances occurred in July and August. AWN-derived temperature threshold exceedances were also detected in the months of October and April, and were most numerous in Klickitat, Benton, and Yakima counties. 

##### Heatwave Days and Employment Days at Risk (AWN)

**Figure 5:**
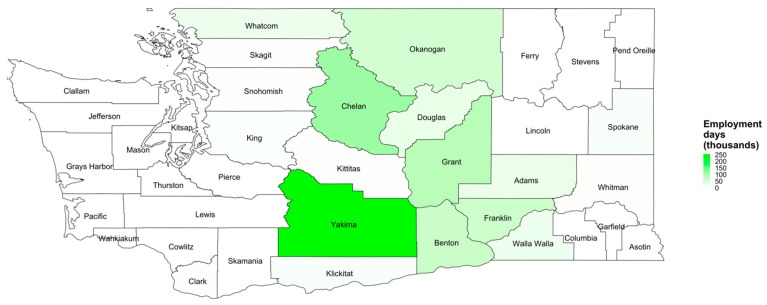
Map of the annual average total employment days (thousands) affected by AWN-derived heatwave days for crop and crop support by county from May to September, 2011–2020. Note: San Juan County and Island County are not labeled.

**Figure A7:**
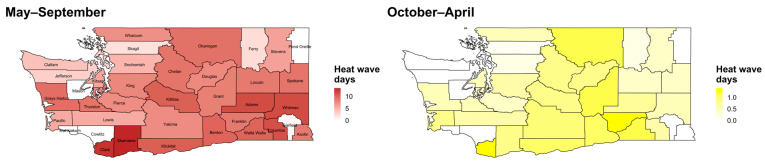
Maps of the annual average number of AWN-derived heatwave days, May–September and October–April, 2011–2020. Note: San Juan County and Island County are not labeled.

Maps of the annual average number of AWN-derived heatwave days for May–September and October–April from 2011 to 2020 are shown in Figure A7, suggesting a less defined delineation of exposure risk between Central and Western WA than that of heat threshold exceedances. The average duration of consecutive heatwave days was 1.8 days (SD = 0.9, range = 1–5 days) among all WA counties in May–September from 2011 to 2020. Generally, counties with the highest average of consecutive heatwave days were found in Central WA (five days), while average successive heatwave day durations of less than five days were observed in coastal counties. The highest number of consecutive heatwave days was a total of five days in June of 2013 in Benton County. The total number of crop employment days affected by AWN-derived heatwave days by county from May to September is shown in Figure 5 and Table S9. Yakima county had the highest annual average total of employment days impacted by heatwave days (254,025 employment days). 

## 4. Discussion

In this county-level descriptive analysis, we found considerable spatiotemporal variability in heat exposure, crop and construction employment, and employment days at risk to heat exposure across Washington State. Overall, there were more days with daily maximum temperatures exceeding thresholds in Central WA, which has substantial agricultural employment, particularly during the summer season, than in Western WA. Crop and construction employment days with daily maximum temperatures exceeding thresholds were highest in counties with large numbers of threshold exceedance days and/or employment (e.g., Yakima County in Central WA and King County in Western WA). Though the majority of days at or above 80 °F (26.7 °C) were between May and September, employment days in April and October at or above 80 °F were apparent in certain counties, especially those in Central WA. These findings provide information that can be used, in combination health outcome data and other information, in heat rule decisions about the timing of provisions, thresholds, and heatwaves. These findings can also inform implementation of heat rule provisions and tailoring of best practices in different industries and geographical areas. Given recent momentum in heat policy development in the U.S. [10,14], it is timely to share data-driven approaches and insights, such as those from this work, that could be adapted in other settings and jurisdictions.

### 4.1. Implications for Heat Rule Decision-Making

Our analyses provide information relevant to decisions about heat rule timing. While the current WA heat rule is in effect at trigger temperatures (52 °F (11.1 °C) for nonbreathable clothes, 77 °F (25.0 °C) for double-layer clothing, 89 °F (31.7 °C) for all other clothing) from May to September [24,25], OR and CA heat rules apply at their respective triggers of a HI of 80 °F and a dry air temperature of 80 °F (26.7 °C) year-round, for all covered workers [11,26]. Our findings in April and October of exceedances of an 80 °F (26.7 °C) threshold in WA, corresponding employment days of exposure, and heatwave days, suggest heat exposure risk outside the May–September window. This is consistent with WA workers’ compensation HRI claims data. An analysis of WA agricultural and forestry WA State Fund workers’ compensation HRI claims reported 95% of HRI claims occurring between May and September with a small proportion of claims occurring in April [45]. A more recent analysis of WA State Fund workers’ compensation data from all sectors, using an HRI case definition from Hesketh et al. [2], indicates approximately 2% of WA State Fund workers’ compensation accepted HRI claims from 2006 to 2021 occurring in April and 0.5% occurring in October [46]. The larger proportion of HRI claims in April than October may reflect HRIs among newer workers who are not fully acclimatized. Overall, our findings suggest that some outdoor crop and construction workers may be at risk for HRI outside the May–September period, especially in April and October, and the distribution of these workers varies by county across WA. These workers could benefit from protections as early as April, which has implications for the optimal timing of training and other heat stress management best practices. Indoor environmental heat exposure conditions may differ from the outdoor ambient environment, and so timing considerations for indoor versus outdoor heat rule provisions may be different.

There are several considerations for decision-making about heat metrics. WBGT, which in a recent analysis has been reported to have a high potential to reflect physiological strain experienced by workers [47], is the basis for WA heat rule trigger temperatures [27] but is not used as an outward-facing metric in existing U.S. heat rules, as it may not be practical to assess in all workplace settings. Though certain groups of workers, such as agricultural workers, may transit between the Western states of CA, OR, and WA for work, these states currently use different metrics (air temperature in CA and WA versus HI in OR) in their heat rules. Some rules (e.g., OR) [11] apply both indoors and outdoors, while others (e.g., CA, WA) only apply outdoors [24,25,26]. Consideration of dew point variability in geographical areas corresponding to employment should be considered in the selection of heat exposure metrics. In a post hoc analysis of dew points of PRISM data from 2002 to 2020 across all WA counties, in which daily mean dew points for each 4 km pixel were averaged over each county and then averaged over each month, we found that the daily mean (standard deviation) dew point in the months of May–September ranged from 32.2 °F (0.1 °C) in Chelan County, a dry Central WA county high in crop worker employment, in May to 53.9 °F (12.2 °C) in Island County, a moist coastal island with minimal outdoor employment, in August. These differences in humidity within a state illustrate the challenges of defining outdoor heat exposure metrics for statewide policies. HI is a simplified metric that accounts for the impact of humidity on worker exposure, yet HI does not take into account solar radiation, makes assumptions about wind level, and may not optimally reflect physiological strain experienced by workers [47]. Considerations of the effect of humidity on HRI risk given the local climate, employment patterns across space and time, the tradeoffs of assessing real-time and forecasted heat exposure metrics, and alignment with nearby jurisdictions where workers may also work should be considered in decisions about heat metrics. Indoor environments, where humidity may be more variable and weather data less applicable, should be considered separately from outdoor environments. 

There are several science-based considerations relevant to establishing trigger temperatures. In addition to the ACGIH-based approach previously used in WA [27], studies suggest an approach that establishes a potential exposure cut point above which HRI cases occur, with this cut-point as a proposed threshold. For example, based on a systematic review of work-related HRI fatalities in which almost all deaths occurred at a HI ≥80 °F (26.7 °C), 80 °F has been recommended as an initial trigger for hazard awareness and protective actions [48]. In WA, workers’ compensation State Fund claims data indicate that 45% of HRI claims occurred below the current WA heat rule trigger of 89 °F (31.7 °C) from 2006 to 2017 [2]. Our study provides employment-day estimates for crop and construction workers in different counties in WA and for different potential thresholds that could be used in required cost–benefit analyses to evaluate potential costs of heat rule provisions and benefits related to prevented HRI, heat-related traumatic injuries [6,7], and acute kidney injury [4,49], and improved productivity [50]. These estimates should be refined with additional local information, for example, from worker and employer surveys, on existing work-shift timing and practices. They should also consider further weather station and employment data analyses of the estimated number of employment days and hours exceeding thresholds if work times are able to be shifted to cooler parts of the day, by subsector. Work times may be more easily shifted in agriculture, for example, during intensive work periods such as harvest, than in construction, where noise ordinances and other process constraints may apply [6]. Scheduling of physically demanding outdoor work tasks, if possible, on cooler days should also be considered.

Secondary analyses with agricultural weather stations allowed us to further explore heat exposure relevant to crop workers in time and space. There are many different networks of weather stations, including those operated by the National Weather Service and private networks such as Weather Underground that have the potential to provide worker-relevant temperature exposure data. However, by using weather stations specifically positioned near agricultural workers (e.g., AWN), we explored the heat exposure and potential HRI burden estimation compared to modeled data, tailored for the crop and crop support subsectors. As the weather-related exposures may vary on a small geographic scale, estimates of exposure are best measured as close to worker populations as possible to minimize potential exposure misclassification. Without information on the location of worker populations within a county, county-level exposure estimates via PRISM must be obtained by averaging all 4 km pixels within a county. Conversely, AWN weather stations are positioned near agricultural operations and may provide a more accurate estimate of exposure if workers are working near AWN stations. For example, in Yakima County, where PRISM modeled data estimated fewer exceedance days than AWN over the study duration, PRISM grids may not fully represent worker exposure variability due to differences in elevation and climate within and among grids, from hot arid river valleys where the majority of crop work occurs to high-elevation mountain slopes lacking workers. Thus, knowledge of worker distributions within a county and the utilization of exposure estimates that best align with these locations may lead to more accurate estimates of the potential burden of HRI.

In addition to newly assigned employees and employees returning from a prolonged absence, employees exposed to suddenly higher temperatures, such as those subject to a heatwave, as defined in the CA heat rule [26], may not be fully acclimatized to the new conditions and need additional protections to reduce the risk of HRI. Reductions in work pace and additional cool-down breaks can reduce time-weighted average heat exposures to safer levels [16]. Agriculturally intensive areas of Central WA had a high number of heatwave employment days and a longer duration of consecutive heatwave days. It should be noted that if conditions plateau at a hot temperature after consecutive heatwave days, the subsequent plateau days may not meet the heatwave definition, as the definition requires a change in temperature from previous days. However, cumulative heat exposure may also increase the risk of HRI and heat-related traumatic injuries. A previous study during the Deepwater Horizon disaster cleanup reported evidence of a cumulative effect on HRI risk from the prior day’s maximum WBGT [51]. A prior study of heat exposure and traumatic injury risk identified an increased risk of injury with cumulative two-day and three-day moving average daily maximum temperatures [52]. Further work is needed to develop an acceptable, reliable, and accurate system for calculating and communicating information about heatwave days to at-risk workers and employers in real time, for conveying information in the form of the different heat metrics used in different heat policies, and for providing information about the location of the heat exposure measurements relative to workers.

### 4.2. Considerations for Other Settings and Jurisdictions

Our analyses have implications for other settings and jurisdictions in which they may be applied. First, our analyses highlight spatiotemporal variability in heat exposure and employment. Rather than evaluating heat exposure and employment at one time point in a small number of locations, data across larger time frames and relevant geographical areas should be examined, with care given to the averaging period and area. Second, the source of heat exposure data must be considered in relationship to the decision or question being examined, as different data sources have different strengths and limitations, especially in the context of outdoor worker populations that are enumerated at a spatial scale that may not match that of estimated worker exposure. We used gridded meteorological data to address questions of time of year and heat exposure thresholds. PRISM data are downloadable and provide a moderate degree of spatial resolution, but these data may not reflect microclimates at smaller spatial scales where certain workers are present. Conversely, weather stations may have more accurate location-specific estimates of exposures and may be located in close geographic proximity to outdoor workers, such as is the case with AWN stations for crop workers. However, weather station data that are summarized over a geographical area should be interpreted with caution, as gaps in weather station spatial distributions may lead to location-specific estimates of exposure being applied to large geographic areas with sparse weather station data. As such, the accuracy of worker exposure estimation may vary from more accurate in regions with higher spatial density of weather stations to less accurate in regions with lower density of weather stations. For example, the probability of detecting heat threshold exceedances or heatwave days is lower in areas where weather stations are sparse, in comparison to areas with more robust weather station coverage. In addition, if weather stations are located at varying elevations within a county, including higher/cooler locations and lower/warmer locations, this could produce different results than if weather stations are clustered in hot or cool areas of a county. Third, employment data must be interpreted with the limitations in the employment data source in mind due to variability in employment reporting practices among and within industries and potential co-occurring variability in exposure estimation.

An additional consideration for other settings and jurisdictions is that data from multiple data sources can provide a more comprehensive picture for decision making than a single source. Heat exposure data alone do not capture worker-relevant exposures without overlaying employment data. However, analyses of environmental exposure and employment do not account for other important risk factors for HRI, including workload, clothing, existing workplace controls, and individual factors. Therefore, if possible, these data should be examined in conjunction with health outcome data, such as workers’ compensation HRI claims data. Given under-recognition and other limitations [2], however, workers’ compensation data are not comprehensive and should be examined in conjunction with other complementary analyses such as those reported in this paper. Further analysis of workers’ compensation data, including categorization of HRI claims by environmental conditions on the day of illness at or above different thresholds, on days with large changes in temperature, and for cumulative exposures, should be conducted. Finally, the implementation of rules and best practices need to be tailored to different industries and populations in different settings and locations and at different times. This tailoring should consider science-based guidance such as that from NIOSH and ACGIH, the scientific literature on the effectiveness of interventions in different industries and settings, and local industry and worker needs. For example, H-2A and other workers may fear reprisal for reporting HRI symptoms and experience transportation, language, and cultural barriers to training and healthcare, which can impact prevention and recovery from heat stress [53]. Additional protections should be implemented for these disproportionately affected workers.

### 4.3. Strengths and Limitations

Our analyses were intended to provide a high-level approach to addressing policy-relevant questions that could be adapted by policymakers for decision-making in other states and jurisdictions, with suggestions for more refined and tailored approaches provided in the discussion. This approach has several strengths and limitations. A strength of our analysis is that the process was driven by common heat policy decision-making questions. Furthermore, we employed validated modeled weather data to estimate exposure in regions that may lack adequate weather station coverage, which was then complemented with data from weather stations in close proximity to agricultural regions where threshold exceedances and crop employment overlaps in time and space. 

Limitations include a lack of direct consideration of certain key heat stress risk factors, including solar radiation, clothing, and workload in the analyses and no consideration of changes in heat exposure projected with climate change. Notably, we do not present measures of WBGT, though the underlying rationale for current permanent WA heat rule triggers are based on WBGT, which provide the most accurate estimate of worker heat stress. County-level analyses also did not consider heat exposure and employment variability within counties or microclimate effects on potential heat exposure related to temperature and/or humidity. Second, the employment data used in these analyses undercount the workforce. For example, BLS QCEW excludes unpaid family members, self-employed workers, and some farm/domestic workers and is estimated to cover only 80 percent of documented farmworker employment [42]. Furthermore, it is estimated that approximately 50% of crop workers may be undocumented [54]. However, QCEW counts jobs and not workers, so workers with multiple jobs can be counted multiple times, which may help to balance this overall underestimation. It should be noted that QCEW estimates require an understanding of state-specific considerations in their interpretation. Another limitation is that we did not consider the day of the week that thresholds were exceeded, or heatwave days occurred, as some workers work on the weekends, and we did not believe it was justified to make systematic assumptions about which days workers work. Finally, though we focused on NAICS subsectors in agriculture that are likely to include outdoor workers (NAICS 111 and 1151), we included all of construction (NAICS 236, 237, and 238). A recent expert review of NAICS construction subsectors in WA in 2022 determined that NAICS codes with a high potential for outdoor work constitute 28.5% of construction workers in WA (including NAICS 237/civil and environmental engineering construction and NAICS 238161/residential roofing contractors) and those with medium potential make up 68.1% of construction workers in WA (including NAICS 236/construction of buildings, NAICS 238212/nonresidential electrical contractors, NAICS 238222/nonresidential plumbing and HVAC contractors, and NAICS 238221/residential plumbing and HVAC contractors) [35].

## 5. Conclusions

We found spatiotemporal variability in heat exposure and crop and construction employment across Washington State. Findings of differential crop and construction employment days above different heat thresholds and for heatwave days over time and space can inform, in combination with health outcome data and other information, decisions about the timing of provisions, thresholds, and heatwaves in occupational heat rule development. Considerations from these analyses are relevant to other settings and jurisdictions and include the selection of heat exposure assessment methods and heat and employment data sources, interpretation of heat and employment data given their unique strengths and limitations and state-specific considerations, use of complementary sources of data, and tailoring of analyses and implementation of policies to specific industries, workers, and environmental contexts. These findings and considerations are timely given projected increases in the frequency and intensity of heat events in the future [28], the recent momentum in heat policy development in the U.S., and the urgent need to protect workers from adverse health effects of heat.

## Data Availability

The data used to generate the findings of this study are publicly available at: PRISM (https://prism.oregonstate.edu; accessed on 1 March 2022), AWN (https://weather.wsu.edu/; accessed 1 March 2022) and BLS QCEW (https://www.bls.gov/cew/downloadable-data-files.htm; accessed on 1 March 2022).

## Supplementary Materials

The following supporting information can be downloaded at: https://www.mdpi.com/article/10.3390/ijerph191811583/s1. Table S1: Annual average number of PRISM derived temperature threshold exceedances, May–September, 2011–2020. Table S2: Annual average number of PRISM derived temperature threshold exceedances, October–April, 2011–2020. Table S3: Annual average (monthly) employment (BLS), May–September, 2011–2020. Table S4: Total annual average employment-days of exposure to temperature threshold exceedances (PRISM), May–September, 2011–2020. Table S5: Construction annual average employment-days of exposure to temperature threshold exceedances (PRISM), May–September, 2011–2020. Table S6: Crop and crop support annual average employment-days of exposure to temperature threshold exceedances (PRISM), May–September, 2011–2020. Table S7: Annual average number of AWN derived temperature threshold exceedances, May–September, 2011–2020. Table S8: Annual average number of AWN derived temperature threshold exceedances, October–April, 2011–2020. Table S9: Annual average total employment-days affected by AWN-derived heat wave days for crop and crop support by county from May–Sept, 2011–2020.
