# Supplementary material for: Potential Impacts of Different Occupational Outdoor Heat Exposure Thresholds among Washington State Crop and Construction Workers and Implications for Other Jurisdictions
Source: Int J Environ Res Public Health. 2022 Sep 14;19(18):11583. doi: 10.3390/ijerph191811583 (PMC9517246; doi:10.3390/ijerph191811583)
Supplement: Supplementary file 1 [file ijerph-19-11583-s001.zip › ijerph-1844532-supplementary.pdf]

SUPPLEMENTAL MATERIAL

Table S1. Annual average number of PRISM derived temperature threshold exceedances, May-September, 2011-2020

County	80ºF (26.7ºC)	85ºF (29.4ºC)	89ºF (31.7ºC)	90ºF (32.2ºC)	95ºF (35.0ºC)
**Benton**	92.6	64.0	42.8	37.8	16.7
**Franklin**	91.4	63.7	41.2	35.7	15.9
**Walla Walla**	86.8	60.7	39.5	34.3	14.4
**Grant**	83.9	56.2	35.0	30.0	12.7
**Adams**	80.5	53.8	32.5	29.1	12.5
**Whitman**	70.5	48.0	29.2	25.0	10.0
**Lincoln**	69.7	43.0	25.9	22.9	7.3
**Douglas**	68.2	41.8	25.5	21.3	6.7
**Spokane**	63.6	38.5	22.4	19.5	4.7
**Asotin**	63.4	41.9	23.8	20.8	50
**Garfield**	63.0	40.2	23.0	19.2	5.0.
**Klickitat**	60.4	34.4	20.2	17.2	5.1
**Columbia**	59.3	34.4	19.2	16.2	3.7
**Stevens**	58.1	34.7	18.7	15.2	3.4
**Ferry**	48.6	25.6	11.6	8.6	1.3
**Yakima**	47.3	23.6	11.2	8.5	0.6
**Clark**	45.2	24.2	10.8	8.7	2.2
**Pend Oreille**	41.9	20.5	7.8	5.4	0.6
**Thurston**	36.8	18.5	7.6	5.7	1.1
**Kittitas**	34.7	15.8	5.8	4.6	0.1
**Cowlitz**	34.1	15.3	6.8	5.5	0.7
**Lewis**	33.3	14.7	6.2	5.0	0.6
**Okanogan**	31.5	13.7	4.3	2.9	0.1
**Mason**	27.2	11.1	4.0	2.8	0.2
**Skamania**	26.8	11.3	3.5	3.0	0
**Kitsap**	26.7	9.7	3.1	2.0	0.1
**Chelan**	24.3	9.5	2.5	1.1	0
**Wahkiakum**	20.1	8.1	3.8	2.7	0.4
**King**	19.6	6.0	1.4	0.8	0

**Pierce**	18.9	5.7	1.3	0.8	0
**Grays Harbor**	16.2	6.2	2.3	1.6	0.1
**Snohomish**	15.9	4.3	0.4	0.2	0
**Pacific**	14.3	5.7	2.3	1.7	0.1
**Skagit**	13.3	2.6	0.1	0.1	0
**Jefferson**	10.0	2.6	0.4	0	0
**Whatcom**	10.0	1.4	0	0	0
**Clallam**	8.1	2.2	0	0	0
**Island**	5.2	0.3	0	0	0
**San Juan**	4.9	0.1	0	0	0
**Median (All counties)**	34.7	15.8	6.8	5.4	0.6
**Standard Deviation (All counties)**	26.4	19.8	13.2	11.7	5.0

Table S2. Annual average number of PRISM derived temperature threshold exceedances, October-April, 2011-2020

County	80°F (26.7°C)	85°F (29.4°C)	89°F (31.7°C)	90°F (32.2°C)	95°F (35.0°C)
**Franklin**	2.1	0.6	0	0	0
**Benton**	2.0	0.4	0	0	0
**Walla Walla**	1.6	0.3	0	0	0
**Grant**	1.5	0.2	0	0	0
**Clark**	1.4	0.2	0	0	0
**Adams**	1.3	0.1	0	0	0
**Whitman**	1.3	0	0	0	0
**Asotin**	0.8	0.1	0	0	0
**Lincoln**	0.8	0	0	0	0
**Garfield**	0.7	0	0	0	0
**Klickitat**	0.7	0	0	0	0
**Spokane**	0.7	0	0	0	0
**Columbia**	0.5	0	0	0	0
**Douglas**	0.5	0	0	0	0

**Thurston**	0.5	0.1	0	0	0
**Wahkiakum**	0.5	0.2	0	0	0
**Lewis**	0.4	0	0	0	0
**Cowlitz**	0.3	0	0	0	0
**Grays Harbor**	0.3	0	0	0	0
**Kitsap**	0.3	0.1	0	0	0
**Mason**	0.3	0	0	0	0
**Pacific**	0.3	0	0	0	0
**Stevens**	0.3	0	0	0	0
**Yakima**	0.2	0	0	0	0
**King**	0.1	0	0	0	0
**Pierce**	0.1	0	0	0	0
**Chelan**	0	0	0	0	0
**Clallam**	0	0	0	0	0
**Ferry**	0	0	0	0	0
**Island**	0	0	0	0	0
**Jefferson**	0	0	0	0	0
**Kittitas**	0	0	0	0	0
**Okanogan**	0	0	0	0	0
**Pend Oreille**	0	0	0	0	0
**San Juan**	0	0	0	0	0
**Skagit**	0	0	0	0	0
**Skamania**	0	0	0	0	0
**Snohomish**	0	0	0	0	0
**Whatcom**	0	0	0	0	0

Table S3. Annual average monthly employment (BLS), May-September, 2011-2020

County	Total	County	Construction	County	Crop and crop support
**King**	62800	**King**	62147	**Yakima**	33012
**Yakima**	35228	**Pierce**	18695	**Chelan**	12145
**Pierce**	19011	**Snohomish**	17526	**Grant**	10223

**Snohomish**	18228	**Clark**	10642	**Franklin**	7254
**Chelan**	13232	**Spokane**	9151	**Benton**	6214
**Grant**	11109	**Whatcom**	3913	**Okanogan**	6104
**Clark**	10886	**Benton**	3346	**Douglas**	3508
**Spokane**	10009	**Thurston**	3299	**Whatcom**	3127
**Benton**	9560	**Skagit**	2829	**Adams**	2494
**Franklin**	8201	**Kitsap**	2801	**Walla Walla**	2007
**Whatcom**	7040	**Yakima**	2216	**Skagit**	1770
**Okanogan**	6463	**Chelan**	1087	**Spokane**	858
**Skagit**	4600	**Cowlitz**	985	**Snohomish**	702
**Douglas**	3829	**Franklin**	948	**King**	653
**Thurston**	3502	**Grant**	886	**Klickitat**	566
**Kitsap**	2857	**Lewis**	839	**Whitman**	386
**Adams**	2527	**Clallam**	762	**Pierce**	315
**Walla Walla**	2357	**Grays Harbor**	679	**Kittitas**	258
**Cowlitz**	1159	**Kittitas**	570	**Clark**	244
**Lewis**	1025	**Island**	560	**Thurston**	203
**Kittitas**	828	**San Juan**	539	**Lincoln**	196
**Clallam**	806	**Okanogan**	360	**Lewis**	186
**Klickitat**	725	**Walla Walla**	350	**Cowlitz**	174
**Grays Harbor**	693	**Mason**	322	**Columbia**	107
**Island**	564	**Douglas**	320	**Kitsap**	56
**San Juan**	556	**Jefferson**	315	**Mason**	49
**Whitman**	548	**Asotin**	240	**Garfield**	44
**Mason**	371	**Whitman**	162	**Clallam**	44
**Jefferson**	337	**Klickitat**	159	**Skamania**	38
**Asotin**	263	**Stevens**	139	**Asotin**	23
**Lincoln**	222	**Pacific**	81	**Jefferson**	22
**Stevens**	140	**Pend Oreille**	50	**San Juan**	17
**Columbia**	116	**Adams**	33	**Grays Harbor**	15
**Pacific**	96	**Lincoln**	26	**Pacific**	15
**Pend Oreille**	61	**Ferry**	12	**Pend Oreille**	10
**Skamania**	50	**Skamania**	12	**Island**	4
**Garfield**	44	**Columbia**	8	**Stevens**	1
**Ferry**	12	**Wahkiakum**	2	**Ferry**	0
**Wahkiakum**	2	**Garfield**	0	**Wahkiakum**	0

Table S4. Total annual average employment-days of exposure to temperature threshold exceedances (PRISM), May-September, 2011-2020

County	80°F (26.7°C)	85°F (29.4°C)	89°F (31.7°C)	90°F (32.2°C)	95°F (35.0°C)
**Yakima**	1737860	880572	416913	317429	23223
**King**	1278498	399755	91851	55043	0
**Grant**	962783	644216	406314	350473	151458
**Benton**	904258	623112	415205	369120	163927
**Franklin**	777633	542711	352319	306795	136583
**Spokane**	645000	391171	225007	196537	47398
**Clark**	504102	270157	123196	99751	25506
**Chelan**	382273	151797	38587	16360	0
**Pierce**	374505	115302	25607	16459	0
**Snohomish**	308033	83814	7950	3832	0
**Douglas**	285127	181276	114213	95525	31098
**Okanogan**	235648	104830	31742	20967	754
**Adams**	212679	142824	85864	77073	33388
**Walla Walla**	205220	143925	92411	78800	31879
**Thurston**	132553	67090	28121	20863	3868
**Whatcom**	83464	11464	0	0	0
**Kitsap**	77999	28903	9376	5892	350
**Skagit**	67632	13117	560	560	0
**Whitman**	42226	29163	17687	15174	6216
**Klickitat**	40451	21776	12126	10241	2945
**Cowlitz**	40015	18136	8148	6529	865
**Lewis**	34787	15469	6680	5330	647
**Kittitas**	31041	14473	5214	4172	94
**Lincoln**	17050	10800	6534	5829	1807
**Asotin**	16682	11195	6632	5854	1346
**Grays Harbor**	11327	4452	1649	1130	78
**Mason**	10584	4432	1623	1126	66
**Stevens**	8163	4894	2658	2154	490

**Columbia**	8024	4649	2604	2212	526
**Clallam**	6803	1907	0	0	0
**Garfield**	3749	2540	1474	1235	335
**Jefferson**	3433	961	167	0	0
**Island**	3001	192	0	0	0
**San Juan**	2872	70	0	0	0
**Pend Oreille**	2666	1379	546	400	55
**Pacific**	1452	593	238	172	10
**Skamania**	1321	519	149	121	0
**Ferry**	599	315	148	113	20
**Wahkiakum**	30	9	3	2	1

Table S5. Construction annual average employment-days of exposure to temperature threshold exceedances (PRISM), May-September, 2011-2020

County	80ºF (26.7ºC)	85ºF (29.4ºC)	89ºF (31.7ºC)	90ºF (32.2ºC)	95ºF (35.0ºC)
**King**	1266123	396079	90977	54584	0
**Spokane**	591044	358649	206507	180159	43507
**Clark**	493623	264619	120744	97701	24953
**Pierce**	367850	113155	25181	16154	0
**Benton**	311118	214599	142880	126618	56090
**Snohomish**	295987	80482	7634	3675	0
**Thurston**	125038	63354	26733	19790	3668
**Yakima**	106945	54396	26202	20003	1356
**Franklin**	86795	60344	38663	33307	14597
**Kitsap**	76501	28309	9183	5775	334
**Grant**	75285	50475	31073	26621	11289
**Whatcom**	40192	5796	0	0	0
**Skagit**	39530	7780	315	315	0
**Cowlitz**	34124	15363	7007	5633	753
**Walla Walla**	31416	22408	14901	13051	5434
**Lewis**	28684	12883	5648	4506	534
**Chelan**	26861	10604	2885	1362	0

**Douglas**	22101	13509	8178	6820	2181
**Kittitas**	20444	9423	3516	2819	58
**Asotin**	15028	10078	5982	5285	1207
**Whitman**	11883	7995	4792	4113	1679
**Okanogan**	11630	5051	1596	1093	36
**Grays Harbor**	11116	4362	1612	1103	73
**Klickitat**	9739	5546	3262	2774	818
**Mason**	8969	3733	1350	935	62
**Stevens**	8123	4872	2646	2146	490
**Clallam**	6417	1775	0	0	0
**Jefferson**	3207	878	148	0	0
**Island**	2952	185	0	0	0
**San Juan**	2773	68	0	0	0
**Adams**	2672	1798	1060	957	424
**Pend Oreille**	2172	1117	453	340	46
**Lincoln**	1751	1061	611	532	153
**Pacific**	1213	499	212	153	10
**Ferry**	599	315	148	113	20
**Columbia**	596	231	108	79	29
**Skamania**	360	165	57	49	0
**Wahkiakum**	30	9	3	2	1
**Garfield**	0	0	0	0	0

Table S6. Crop and crop support annual average employment-days of exposure to temperature threshold exceedances (PRISM), May-September, 2011-2020

County	80°F (26.7°C)	85°F (29.4°C)	89°F (31.7°C)	90°F (32.2°C)	95°F (35.0°C)
**Yakima**	1630916	826176	390711	297426	21868
**Grant**	887498	593742	375241	323851	140169
**Franklin**	690838	482367	313656	273488	121986
**Benton**	593140	408514	272324	242502	107837
**Chelan**	355412	141193	35703	14998	0
**Douglas**	263026	167767	106034	88705	28917

**Okanogan**	224018	99779	30146	19874	718
**Adams**	210007	141026	84804	76116	32964
**Walla Walla**	173804	121517	77510	65749	26445
**Spokane**	53957	32522	18501	16377	3892
**Whatcom**	43272	5668	0	0	0
**Klickitat**	30712	16230	8864	7467	2127
**Whitman**	30344	21168	12896	11062	4537
**Skagit**	28102	5337	245	245	0
**Lincoln**	15299	9739	5923	5297	1653
**King**	12375	3676	874	459	0
**Snohomish**	12046	3331	317	157	0
**Kittitas**	10598	5050	1698	1353	36
**Clark**	10479	5538	2452	2050	554
**Thurston**	7515	3736	1388	1073	200
**Columbia**	7428	4418	2496	2133	498
**Pierce**	6655	2147	426	305	0
**Lewis**	6103	2586	1032	824	113
**Cowlitz**	5891	2773	1141	896	111
**Garfield**	3749	2540	1474	1235	335
**Asotin**	1654	1117	650	570	139
**Mason**	1616	699	273	192	4
**Kitsap**	1497	594	193	117	16
**Skamania**	961	355	91	72	0
**Pend Oreille**	493	263	94	60	9
**Clallam**	385	132	0	0	0
**Pacific**	239	94	26	19	0
**Jefferson**	226	84	19	0	0
**Grays Harbor**	211	90	36	27	5
**San Juan**	100	3	0	0	0
**Island**	49	7	0	0	0
**Stevens**	40	22	12	8	0
**Ferry**	0	0	0	0	0
**Wahkiakum**	0	0	0	0	0

Table S7. Annual average number of AWN derived temperature threshold exceedances, May-September, 2011-2020

County	80°F (26.7°C)	85°F (29.4°C)	89°F (31.7°C)	90°F (32.2°C)	95°F (35.0°C)
**Franklin**	102.5	74	50.2	43.4	19.4
**Benton**	97.8	70.2	48.5	42.4	18.7
**Walla Walla**	97.6	71.5	49.5	43.1	18.9
**Okanogan**	96.2	70.5	47.5	41.7	18.5
**Klickitat**	96.0	70.7	46.5	40.9	18.9
**Yakima**	94.6	67.7	42.7	37.3	14.3
**Grant**	90.2	62.4	37.0	32.0	13.0
**Adams**	89.9	63.8	40.0	35.3	17.2
**Chelan**	88.5	64.0	41.8	35.2	15.4
**Douglas**	87.5	62.8	39.8	34.6	14.0
**Columbia**	77.1	53.7	32.9	29.0	11.8
**Whitman**	75.4	49.7	30.8	26.5	10.8
**Kittitas**	75.2	48.6	26.6	23.4	7.9
**Spokane**	70.4	43.5	25.5	22.1	5.7
**Lincoln**	68.3	40.7	24.7	21.5	6.4
**Skamania**	64.5	36.4	19.5	17.2	4.8
**Asotin**	61.8	36.2	20.5	16.3	2.8
**Clark**	61.1	33.0	16.7	13.6	3.9
**Stevens**	51.9	35.9	23.5	20.2	9.2
**Thurston**	49.7	26.7	13.3	10.4	2.3
**Pierce**	46.2	22.2	7.9	6.1	0.9
**King**	44.8	20.4	8.2	6.2	0.3
**Whatcom**	36.0	13.3	4.8	3.5	0.2
**Kitsap**	32.3	14.3	5.0	3.4	0.2
**Ferry**	30.9	21.2	11.4	9.3	2.5
**Snohomish**	26.7	8.6	1.9	0.7	0.1
**Lewis**	25.6	13.7	7.2	6.1	1.7
**Grays Harbor**	14.7	6.4	2.6	1.9	0.1
**Jefferson**	13.0	5.4	0.6	0.2	0
**Skagit**	13.0	1.7	0.3	0.2	0

**Clallam**	11.5	3.3	0.3	0.3	0
**Island**	11.5	2.9	0.3	0.2	0
**Pacific**	6.2	3.7	1.4	1.1	0.1
**Garfield**	5.9	3.5	1.9	1.7	0.8
**San Juan**	0	0	0	0	0
**Mason**	0	0	0	0	0
**Cowlitz**	0	0	0	0	0
**Pend Oreille**	0	0	0	0	0
**Wahkiakum**	0	0	0	0	0
**Median (All counties)**	49.7	26.7	13.3	10.4	2.5
**Standard Deviation (All counties)**	35.6	26.6	18.0	15.8	7.1

Table S8. Annual average number of AWN derived temperature threshold exceedances, October-April, 2011-2020

County	80°F (26.7°C)	85°F (29.4°C)	89°F (31.7°C)	90°F (32.2°C)	95°F (35.0°C)
**Klickitat**	1.4	0.3	0	0	0
**Benton**	1.1	0.3	0	0	0
**Yakima**	1.1	0.1	0	0	0
**Walla Walla**	1.0	0.3	0	0	0
**Franklin**	0.9	0.2	0	0	0
**Skamania**	0.9	0.1	0	0	0
**Kittitas**	0.8	0.1	0	0	0
**Adams**	0.7	0.0	0	0	0
**Clark**	0.7	0.0	0	0	0
**Chelan**	0.6	0.1	0	0	0
**Grant**	0.6	0.1	0	0	0
**Whitman**	0.6	0.0	0	0	0
**Douglas**	0.4	0.1	0	0	0

**Columbia**	0.4	0.0	0	0	0
**Okanogan**	0.4	0.0	0	0	0
**Pacific**	0.3	0.0	0	0	0
**Grays Harbor**	0.2	0.0	0	0	0
**Spokane**	0.2	0.0	0	0	0
**Asotin**	0.1	0.0	0	0	0
**Lincoln**	0.1	0.0	0	0	0
**Stevens**	0.1	0.0	0	0	0
**Clallam**	0	0.0	0	0	0
**Ferry**	0	0.0	0	0	0
**Garfield**	0	0.0	0	0	0
**Island**	0	0.0	0	0	0
**Jefferson**	0	0.0	0	0	0
**King**	0	0.0	0	0	0
**Kitsap**	0	0.0	0	0	0
**Lewis**	0	0.0	0	0	0
**Pierce**	0	0.0	0	0	0
**Skagit**	0	0.0	0	0	0
**Snohomish**	0	0.0	0	0	0
**Thurston**	0	0.0	0	0	0
**Whatcom**	0	0.0	0	0	0
**San Juan**	0	0.0	0	0	0
**Mason**	0	0.0	0	0	0
**Cowlitz**	0	0.0	0	0	0
**Pend Oreille**	0	0.0	0	0	0
**Wahkiakum**	0	0.0	0	0	0

Table S9. Annual average total employment-days affected by AWN-derived heat wave days for crop and crop support by county from May-Sept, 2011-2020

County	Employment-days at risk
**Yakima**	254025
**Chelan**	111781
**Grant**	85879

**Benton**	68266
**Franklin**	65490
**Okanogan**	56432
**Douglas**	29377
**Adams**	27852
**Whatcom**	23992
**Walla Walla**	23439
**Spokane**	7364
**Klickitat**	6936
**King**	5061
**Whitman**	4312
**Snohomish**	3516
**Skagit**	3436
**Clark**	3047
**Kittitas**	2769
**Pierce**	2224
**Thurston**	1681
**Lincoln**	1672
**Columbia**	1175
**Lewis**	672
**Skamania**	495
**Kitsap**	335
**Clallam**	210
**Asotin**	195
**Grays Harbor**	143
**Jefferson**	95
**Pacific**	73
**Garfield**	23
**Island**	19
**Stevens**	7
**Cowlitz**	0
**Ferry**	0
**Mason**	0

**Pend Oreille**	0
**San Juan**	0
**Wahkiakum**	0
